# Quality Improvement Project (QIP) Bryngolau–Implement and Optimise Non-Pharmacological Approaches for Managing BPSD (Behavioural and Psychological Symptoms of Dementia) to Minimise the Need for Pharmacological Interventions

**DOI:** 10.1192/bjo.2026.11489

**Published:** 2026-06-30

**Authors:** Faryal Sohail, Graham O'Connor, Padmavathy Gopinath, Saima Saleem, Golam Ishraque Chowdhry

**Affiliations:** 1 Hywel Dda Health Board, Llanelli, United Kingdom; 2 Hywel Dda Health Board, Carmarthen, United Kingdom

## Abstract

**Aims:**

A Quality Improvement Project (QIP) aimed to reduce the pharmacological interventions for behavioural and psychological symptoms of Dementia(BPSD) by 40–60% on Bryngolau Ward, a specialist Old Age Psychiatry unit in Prince Philip Hospital, Llanelli; through optimised non-pharmacological strategies.

**Methods:**

Using the Model for Improvement and Plan–Do–Study–Act (PDSA) cycles, the project implemented a combination of interventions including staff education, sensory room utilisation, guideline promotion, and structured assessment tools (e.g., ABC charts). Baseline data (two 15-day periods) were compared against four PDSA cycles (15 days each)measuring BPSD incidence, use of pharmacological and non-pharmacological interventions, and falls.

**Results:**

Pharmacological interventions decreased from 44.3% (baseline) to 23.5% (Cycle 4), exceeding the target (average reduction: 42.5%). Non-pharmacological interventions rose from 55.7% to 76.5%. BPSD incidents dropped by 56% (Cycle 4), and falls reduced by 76% (Cycle 3). Variability in Cycle 3 highlighted situational challenges, but overall trends demonstrated sustained improvement.

**Conclusion:**

The QIP successfully shifted practice towards non-pharmacological BPSD management, reducing medication use and improving patient safety; adhering to national guidelines. Key drivers included staff training, sensory resources, and iterative PDSA refinements. Recommendations include embedding these strategies into standard practice, ongoing staff development, and expanding monitoring to ensure sustainability. This project provides a replicable framework for dementia care settings aiming to minimise pharmacological reliance.

